# Feasibility of Applying Proton Minibeam Technology on the Proton Therapy Complex “Prometheus”

**DOI:** 10.17691/stm2026.18.4.01

**Published:** 2026-08-31

**Authors:** O.Yu. Golovanova, G.V. Merzlikin, A.D. Kaprin, A.N. Soloviev, V.O. Saburov, S.N. Koryakin

**Affiliations:** Engineer-Physicist, Laboratory for the Development and Operation of Irradiation Equipment; A. Tsyb Medical Radiological Research Center — Branch of the National Medical Research Radiological Center, Ministry of Health of the Russian Federation, 4 Koroleva St., Obninsk, 249036, Russia; Engineer, Laboratory for the Development and Operation of Irradiation Equipment; A. Tsyb Medical Radiological Research Center — Branch of the National Medical Research Radiological Center, Ministry of Health of the Russian Federation, 4 Koroleva St., Obninsk, 249036, Russia; Research Intern, Laboratory of Medical Physics; Institute for Nuclear Research of the Russian Academy of Sciences, 7a 60th Anniversary of October Avenue, Moscow, 117312, Russia; MD, DSc, General Director; National Medical Research Center for Radiology, Ministry of Health of the Russian Federation, 3 2nd Botkinsky Proezd, Moscow, 125284, Russia; PhD, Head of the Laboratory of Medical Radiation Physics; A. Tsyb Medical Radiological Research Center — Branch of the National Medical Research Radiological Center, Ministry of Health of the Russian Federation, 4 Koroleva St., Obninsk, 249036, Russia; Associate Professor, Department of Radionuclide Medicine; sObninsk Institute for Nuclear Power Engineering — Branch of the National Research Nuclear University “MEPhI”, 31 Kashirskoe Shosse, Moscow, 115409, Russia; Research Associate, Acting Head of the Laboratory for the Development and Operation of Irradiation Equipment; A. Tsyb Medical Radiological Research Center — Branch of the National Medical Research Radiological Center, Ministry of Health of the Russian Federation, 4 Koroleva St., Obninsk, 249036, Russia; PhD, Head of the Department of Radiation Biophysics; A. Tsyb Medical Radiological Research Center — Branch of the National Medical Research Radiological Center, Ministry of Health of the Russian Federation, 4 Koroleva St., Obninsk, 249036, Russia; Associate Professor, Department of Radionuclide Medicine; Obninsk Institute for Nuclear Power Engineering — Branch of the National Research Nuclear University “MEPhI”, 31 Kashirskoe Shosse, Moscow, 115409, Russia

**Keywords:** radiation therapy, proton therapy, radiochromic films, mathematical modeling, TOPAS MC

## Abstract

Spatial fractionation of proton beams is being considered as a new strategy to reduce the probability of side-effects in radiation therapy on the proton therapy complex (PTC) “Prometheus”. By collimating the beams to submillimeter dimensions, the dose profile at the target surface consists of alternating high- and low-dose regions. This promotes sparing of normal tissues along the beam path through the activation of various biological mechanisms.

**The aim of the study** was to implement the minibeam method on the PTC “Prometheus” and to experimentally assess the quality of the resulting dose distribution.

**Materials and Methods:**

Collimation of the primary beam was achieved using brass multislit collimators. Mathematical modeling of the experiment was performed, and a phantom was irradiated from one and from two opposite directions. For effective summation of two fields and to achieve a homogeneous dose distribution in the target, intensity and energy modulation of the proton beams entering the collimator was carried out. To reduce errors caused by collimator deviation relative to the beam axis, a quality assurance system for collimator positioning on the treatment table was developed. Under the control of a scintillation detector and a laser positioner, positioning accuracy was within 0.2°. GafChromic EBT3 radiochromic films (Ashland, USA) were used as dosimeters.

**Results:**

When comparing calculated and experimental data, 90% of the distribution points matched the mathematical model according to the γ criterion (3%/3 mm). The quality of the minibeam field was assessed using the following parameters: valley-to-peak dose ratio; collimator throughput; ratio of the dose without the collimator to the dose with the collimator; and dose homogeneity index in the target. The obtained results met the quality assurance criteria for standard radiation therapy.

**Conclusion:**

Irradiation with proton minibeams using brass collimators is feasible on the PTC “Prometheus”. A uniform dose distribution in the target area was experimentally achieved. The uniformity of coverage, the absence of local dose excesses, and the high dose gradient at the distal edge suggest that the described irradiation technique can be implemented on biological specimens.

## Introduction

In recent years, the method of proton minibeam radiation therapy (pMBRT) has been actively discussed as a way to reduce the risk of radiation complications during proton therapy [1]. This approach combines the advantages of scanned proton beams with spatial fractionation. Unlike conventional therapy, proton minibeam therapy delivers the entrance dose distribution with submillimeter resolution using collimation, which creates alternating high and low dose regions along the beam path to the target. This dose distribution pattern helps protect healthy tissues through a number of biological mechanisms [2]. The dose distribution becomes more uniform within the target depth due to Coulomb scattering. Experimental studies by Sammer [3, 4] and Prezado [5] confirm that it is possible to safely exceed local tolerance doses for normal tissues and demonstrate an increase in the therapeutic index.

To ensure uniform dose coverage, a treatment scheme using two opposing fields with a partial shift between them has been proposed [6]. This method achieves high homogeneity within the target tumor volume while preserving heterogeneity along the beam path to the target. Such a configuration opens up prospects for dose escalation in the treatment of radioresistant tumors, re-irradiation, and hypofractionated regimens.

**The aim of this study** was to implement the minibeam method on the proton therapy complex (PTC) “Prometheus” and to experimentally assess the quality of the resulting dose distribution.

## Materials and Methods

### Criteria for assessing the quality of spatial fractionation

The main objective of proton minibeam therapy is the efficient generation of proton minibeams. A collimator is used to achieve the intended dose distribution. It consists of a thick metal block with one or more apertures [1, 7]. In addition, it is possible to focus proton beams to submillimeter diameters using magnetic optics [8] or a scanning dynamic collimator [9]. However, given the technical capabilities and the complexity of implementing these methods, a metal collimator remains the most accessible and easily realizable solution.

The quality of spatial fractionation is proposed to be assessed using the following parameters [10]:

valley-to-peak dose ratio;
throughput;
grid factor;
dose homogeneity in the target.

*The valley-to-peak dose ratio* (VPDR) is a dosimetric parameter that reflects the spatial dose heterogeneity. This value ranges from 0 to 1, with a lower value indicating a more pronounced dose contrast between the peaks and valleys [10]. The valley-to-peak dose ratio is expressed as VPDR(*Z*)=*D*_min_/*D*_max_, where *Z* is the tissue depth.

When the dose difference between peaks and valleys is significant, the VPDR value approaches 0. At the same time, a VPDR value of 1 is required within the target region to ensure a uniform dose.

*The throughput* is the ratio of the number of protons reaching the target to the number of primary protons. This parameter describes the collimator’s transmission efficiency. In addition to geometric properties of the collimator, the throughput is affected by various beam parameters, such as transverse beam distribution, beam solid angle determined by the scanning system, and initial beam angular spread [11].

*The grid factor*, proposed by Reaz [10], is defined as the ratio of the dose at the target without the collimator to the dose at the target with the collimator: Grid factor=*D_ref_*/*D_grid_*.

*Dose homogeneity* describes the uniformity of the dose distribution within the target volume. It is important for assessing the therapeutic applicability of the irradiation technique.

The homogeneity index is a measure of the dose coverage uniformity of the target volume. It is calculated as the difference between the doses received by 2% and 98% of the tumor volume (D_2%_–D_98%_), divided by the median dose (D_50%_) [12]. In this research, the acceptable range of dose heterogeneity within the intended target volume is defined according to standards for conventional radiotherapy and makes up 95–107% of the prescribed dose. In relative terms, the homogeneity index should not exceed 0.12 [13].

### Experiment

A proton therapy complex (PTC) “Prometheus” (JSC “Protom”, Russia) [14] with a scanned proton beam of energies from 40 to 250 MeV was used as the radiation source.

A multislit collimator was made of brass to generate minibeams. The collimator apertures were made by electrical discharge machining as five parallel slits 0.4 mm wide and 20 mm high, with a center-to-center spacing of 4 mm. The collimator thickness was 65 mm, which minimized the valley dose and increased the peak-to-valley dose ratio [15].

The irradiation object was a water phantom with a 20×20 mm target located at the center of the tumor volume. The collimator was placed flush against the phantom wall. Irradiation was carried out from two opposite beam directions: 0° and 180°. The entrance fields were identical and symmetrical as the target was in the phantom center. The collimators were shifted relative to each other by half the center-to-center distance to create a homogeneous field within the target. A quality assurance system for collimator positioning on the treatment table was developed to reduce positioning errors caused by collimator misalignment with respect to the beam axis. Under the control of a scintillation detector and a laser positioner, positioning accuracy was within 0.2°.

Two dose distribution configurations are considered: a monoenergetic mode at 100 MeV and a spread-out Bragg peak with a maximum energy of 100 MeV.

#### Irradiation from a single direction

To assess the feasibility of using a longitudinally heterogeneous dose distribution on the PTC “Prometheus”, irradiation with minibeams from a single direction was first considered, both in monoenergetic mode and in spread-out Bragg peak mode [16].

Mathematical modeling of the experiment was done using the TOPAS MC software package [17]. The beam model accurately described the actual PTC beam [11]. The initial beam positions were calculated according to their expected deviation in the plane for which transverse beam dimensions were reliably determined. Beam geometric dimensions and angular spread for the energy range of 80–160 MeV in 10 MeV steps were obtained from physical dosimetric measurements. Data for intermediate energies not obtained by measurements were determined by interpolating experimental data using a fifth-degree polynomial. The resulting parameter file (accelerator control file) contained data on beam displacement angles from the central axis, nominal beam energy, and number of particles at each point.

Verification of the mathematical modeling results was conducted both with and without the collimator. GafChromic EBT3 radiochromic films (Ashland, USA) were used as detectors, following the recommendations of AAPM TG-235 [18] and TRS-483 [19]. One of the challenges in film dosimetry for charged particle beams is the decrease in response with increasing linear energy transfer of the ionizing radiation [20]. Therefore, the measured dose values were normalized to the maximum value. For the experiment, a water phantom measuring 120×65 mm with a 5-mm-thick polymethyl methacrylate entrance window was constructed in accordance with the calculation parameters.

To analyze the transverse beam dose distribution, dose profiles were selected at the phantom entrance (Z=0 mm) and at the center of the spread-out Bragg peak plateau (Z=58 mm). The discrepancy between measured and calculated dose values was assessed using the one-dimensional gamma index (γ). Our team developed a Python program using the math and numpy modules to determine the number of points satisfying the gamma criterion within acceptable dose and depth tolerances. A gamma value of γ< 1 indicated that the point successfully passed the correlation test. In clinical practice, a passing rate of 90% of points meeting the γ (3%/3 mm) criterion is considered acceptable [21].

Films placed in front of the collimator showed good agreement between measured dose values at a point and mathematical modeling results, confirming the reliability of our PTC beam model. Experimental verification demonstrated that the dose coverage on films placed inside the phantom matched the calculated values in a case of single-field irradiation, both in monoenergetic mode and in spread-out Bragg peak mode. The VPDR ratio was 0.02 at Z=0 mm and 0.60 at Z=58 mm. For the spread-out Bragg peak, the VPDR ratio was 0.04 and 0.61, respectively. These data are consistent with values reported in the literature [1] and with our mathematical model, which allowed us to proceed to the next stage of the experiment.

#### Irradiation from two directions

**Figure 1.**
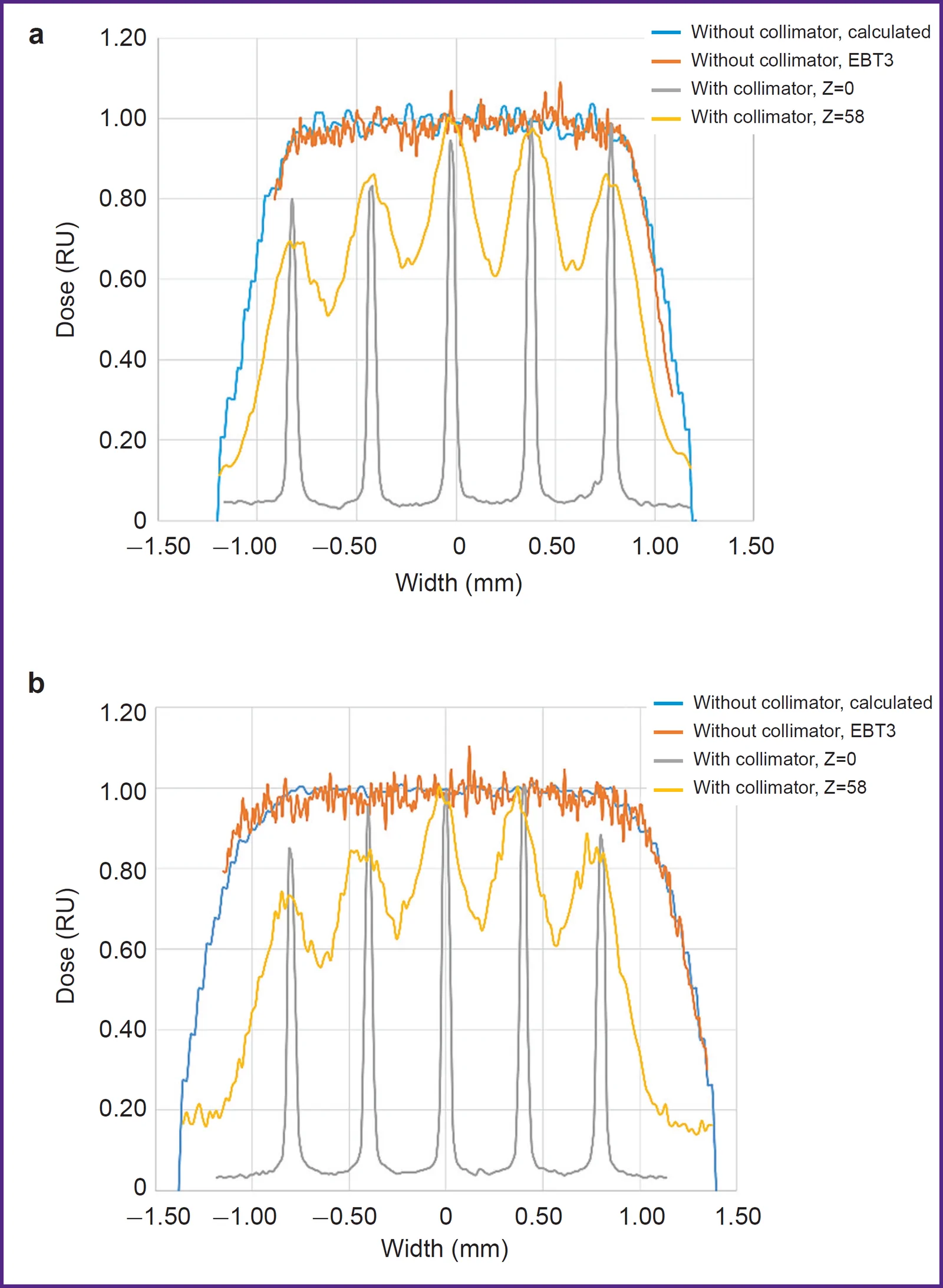
Transverse dose profiles for single-field irradiation without intensity modulation in monoenergetic mode (a) and spread-out Bragg peak mode (b) Mathematical model (*blue*) and dose distribution obtained as a result of irradiation of GafChromic EBT3 radiochromic film (Ashland, USA): without collimator (*orange*), with collimator at depth Z=0 (*gray*), with collimator at depth Z=58 (*yellow*)

As shown in Figure 1, the dose in the outermost minibeams is 20% lower than in the central ones. This effect may be explained by Coulomb scattering of protons in the collimator material and by the fact that the beams have a nonzero solid angle. Our team decided that adjustments to the model were necessary to achieve a homogeneous field in the target region when irradiating from two opposite directions.

Correction of the heterogeneity of the resulting field was made by modulating the number of particles in the primary field. In the computer model of the experimental setup within the TOPAS MC software package, flat detector plates were added for each of the five collimator slits, and the particle fluence after passing through each slit was determined. The obtained values were normalized to unity. Thus, weighting coefficients were determined for groups of beams corresponding to each collimator slit. When developing the irradiation treatment plan, the scanning step size directly depends on the transverse dimensions of the primary beam energy for a group of particles with a fixed energy. These dimensions are not constant and decrease with increasing energy. When forming the spread-out Bragg peak, in addition to weighting coefficients, the specific number of particles of a given energy per unit area is taken into account to compensate for the difference in scanning step size.

## Results

**Figure 2.**
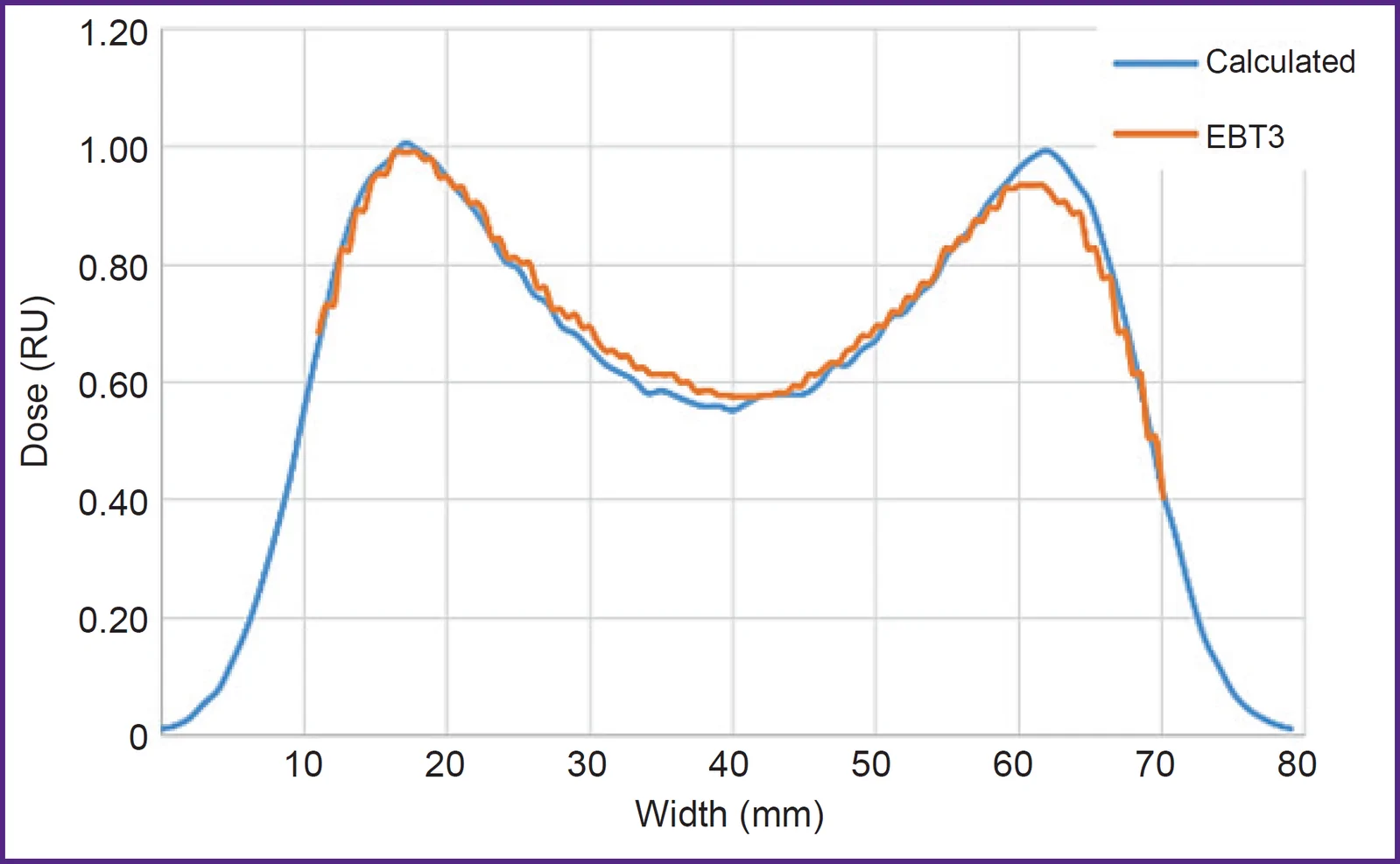
Transverse dose profile of the entrance field with modulated intensity without collimator: mathematical model (*blue*) and measurement result of irradiation of GafChromic EBT3 radiochromic film (Ashland, USA) (*orange*)

The transverse dose profile for the intensity-modulated field before passing through the collimator is shown in Figure 2. When comparing calculated and experimental data, 90% of the profile points matched the mathematical model according to the γ (3%/3 mm) criterion.

**Figure 3.**
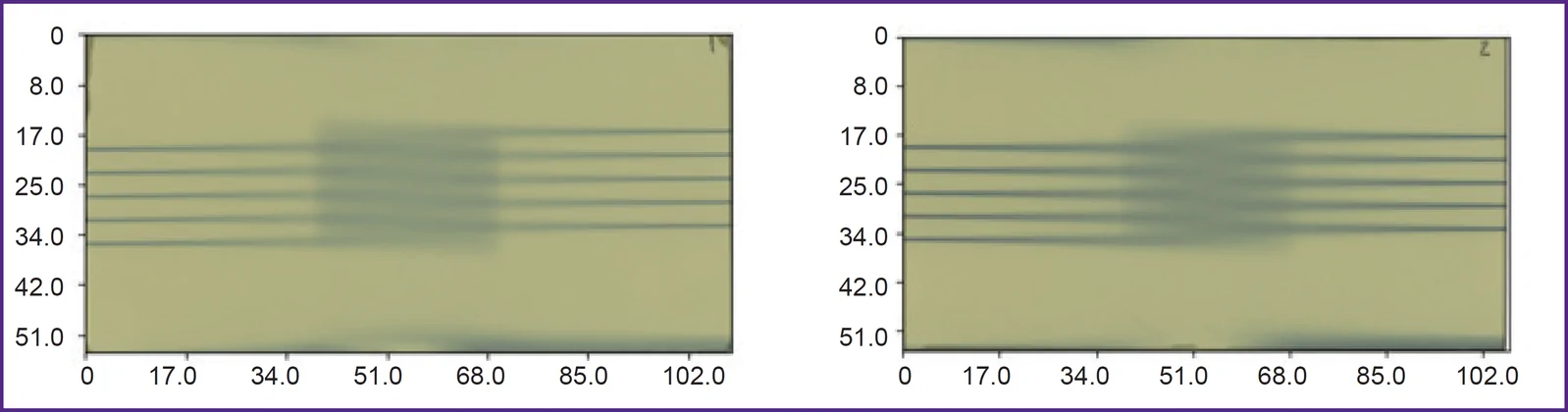
Radiochromic film after irradiation from two directions: in monoenergetic mode (*left*) and spread-out Bragg peak mode (*right*)

**Figure 4.**
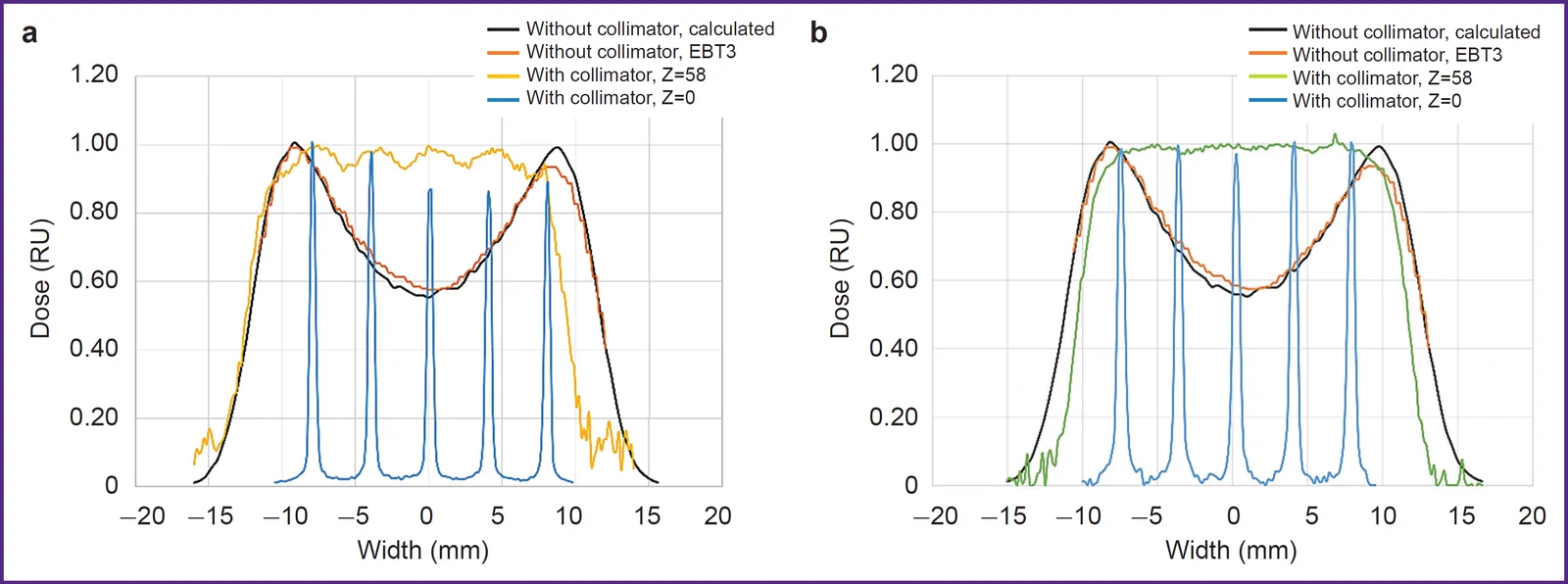
Transverse dose profiles for irradiation with two opposed fields with intensity modulation in monoenergetic mode (a) and spread-out Bragg peak mode (b) Mathematical model of the field without collimator (*black*) and dose distribution obtained from radiochromic film processing: field without collimator (*orange*), with collimator at depth Z=0 (*blue*), with collimator at depth Z=58 (*yellow/green*)

As a result of irradiating the target from two opposite directions with an intensity-modulated field, both in monoenergetic mode and in spread-out Bragg peak mode, the expected dose distribution was obtained (Figure 3). The transverse dose profiles derived from radiochromic film processing are shown in Figure 4.

Dose coverage uniformity was assessed within the target region. The local dose excess due to heterogeneity of the entrance field was 2.62% for the spread-out Bragg peak mode and 3.61% for the monoenergetic mode. The quality of spatial fractionation was also assessed using the following parameters (see the Table): valley-to-peak dose ratio (VPDR); collimator throughput; and ratio of dose without the collimator to dose with the collimator (grid factor).

**Table:** Criteria for spatial fractionation quality

Quality criterion	Monoenergetic mode, 100 MeV		Spread-out Bragg peak mode	
	Z=0 mm	Z=58 mm	Z=0 mm	Z=58 mm
VPDR	0.02	0.60	0.04	0.61
Throughput (%)	55–60	—	52–57	—
Grid factor	—	0.77	—	0.18
Dose homogeneity	—	3.61	—	2.62

## Discussion

In this research, mathematical modeling of the interaction between the scanning beam of the PTC “Prometheus” and the metal collimator was of great importance. The experimental values of the one-dimensional gamma index confirm the reliability of the mathematical model and its applicability for further research.

Studies by foreign authors [1, 10] are generally specific to proton therapy systems manufactured by Varian (USA). For this reason, we were interested in testing the applicability of the parameters proposed by Reaz [10] for assessing the quality of spatial fractionation of a Russian system.

The VPDR values at the beginning of the range were close to 0 — at the phantom entrance, the highest contrast was observed between the dose in the minibeams and the dose between the minibeams as there was no air gap between the collimator and the water phantom wall. At the center of the target, the VPDR was close to 1 but not equal to 1, indicating dose heterogeneity within the target region. The acceptable local dose excess in the target volume varies among different authors [12]. The assessed dose homogeneity within the target did not exceed 4%. These values fall within the tolerance heterogeneity range accepted in our work (95–107% of the prescribed dose) and comply with radiotherapy standards for small irradiation fields [19]. This result was achieved by modulating the entrance field intensity and equalizing the peak dose heights.

The throughput of the brass collimators used in this study was 55–60% and 52–57%, which is consistent with literature data [22]. A correlation was observed between VPDR and collimator throughput. Geometrically, throughput depends on collimator thickness, slit width, and slit spacing. It is important to find a balance between these parameters when spatially fractionating the dose. A thinner collimator allows more protons to pass through the slits, but the dose contrast between minibeams is low. The thicker the collimator is and the narrower the slits are, the fewer protons reach the target. As a result, the dose distribution within the target becomes heterogeneous, even though the VPDR value approaches 0. This raises the question of the minibeam generation efficiency. The magnitude of proton loss due to mechanical collimation is assessed using the grid factor [10]. The ratio of the dose at the target without the collimator to the dose at the target after passing through the collimator in our experiment was 0.77 for the monoenergetic mode and 0.18 for the spread-out Bragg peak mode. A grid factor value approaches 1 in the absence of particle loss due to collimation. We obtained a relatively high grid factor for the monoenergetic mode; the 20–25% dose difference can be compensated by increasing the number of protons in the treatment plan. A substantial loss (about 80%) of primary particles was observed for the spread-out Bragg peak. This result may be explained by differences in the displacement angles of protons of different energies by the scanning system and their absorption in the collimator walls, which requires further research.

High positioning accuracy is required in future work with the metal collimator, since even a 0.2° or 0.1 mm displacement in one of the planes causes a significant change in a field configuration. A greater number of protons remain in the collimator walls, leading to an increase in secondary neutron production. For dealing with biological specimens, it is necessary to assess the dose contribution from secondary neutrons and their impact on the occurrence of adverse effects in radiation therapy.

## Conclusion

Our study confirms the feasibility of generating and applying proton the minibeam method on the PTC “Prometheus”. The brass multislit collimator combined with intensity modulation made it possible to create a uniform dose distribution within the target volume when irradiating from two opposite directions. Comparison of calculated and experimental data showed good agreement, indicating the correctness of the chosen model and the high accuracy of the method. The obtained parameters included homogeneous dose coverage, absence of local dose peaks, and a sharp gradient at the distal edge. They suggest the potential for practical application of this technique in studies with biological specimens. Therefore, the implemented approach can be considered promising for the development of proton therapy aimed at increasing the therapeutic index and reducing the likelihood of side effects.
